# Women as Doctors

**Published:** 1913-08-02

**Authors:** Mary Scharlieb, May Thorne

**Affiliations:** Harley Street, W.; Harley Street, W.


### Women as Doctors.

*To the Editor of* The Hospital.

Sir,—We have read Professor Hochenegg's statement that he considers "that women are not adapted for the work of a medical practitioner." We note that he bases his opinion on his acquaintance with women medical students. We would point out to Professor Hochenegg that his opinion is not shared by medical men in England nor by those in authority in the public services of this country.

For many years women have worked on the mixed staffs of the Royal Free Hospital, London, the Stanley Hospital, Liverpool, the Women's Hospital, Birmingham, the Victoria Hospital for Children, Hull, with mutual satisfaction. A woman doctor is a Government Inspector of Prisons, other medical women hold responsible positions on various public bodies, and the expert experience of women doctors is not only sought by Departmental Committees and Royal Commissions, but in daily work many men practitioners seek the aid of medical women to advise on their patients and to perform major operations.

We should like Professor Hochenegg to read the annual report of the New Hospital for Women, in order that he might learn the nature of the operations successfully undertaken by women surgeons in a hospital staffed by women only. We would suggest to Professor Hochenegg that it is hardly fair to judge of the capabilities of experienced physicians and surgeons when he has only seen women in the early days of their medical studies.—We are, Sir, faithfully yours,

Mary Scharlieb, M.D., M.S.Lond.

May Thorne, F.R.C.S.I., M.D.

Harley Street, W.,
July 29, 1913.

